# An ultra-sensitive and sustainable spectrofluorimetric approach for trace analysis of dinitolmide residues in complex food matrices

**DOI:** 10.1039/d6ra07003e

**Published:** 2026-11-10

**Authors:** Galal Magdy, Shaimaa Ebrahim, Ramadan A. El-Domany, Fathalla Belal

**Affiliations:** a Department of Pharmaceutical Analytical Chemistry, Faculty of Pharmacy, Mansoura National University Gamasa 7731168 Egypt galal_magdy@pharm.kfs.edu.eg +201000137394; b Pharmaceutical Analytical Chemistry Department, Faculty of Pharmacy, Kafrelsheikh University Kafrelsheikh 33511 Egypt; c Department of Microbiology and Immunology, Faculty of Pharmacy, Kafrelsheikh University Kafrelsheikh P. O. Box 33511 Egypt; d Pharmaceutical Analytical Chemistry Department, Faculty of Pharmacy, Mansoura University Mansoura 35516 Egypt

## Abstract

The extensive veterinary use of dinitolmide (DNT) as an anticoccidial drug has raised increasing concerns regarding its residual occurrence in poultry-derived foods and the associated risks to consumer health, necessitating the development of rapid, sensitive, and environmentally sustainable analytical methods. Herein, the first spectrofluorimetric method for the determination of DNT residues in poultry matrices is presented. The assay is based on the fluorescence response observed after treatment of DNT with a Zn/HCl system. This response is consistent with the proposed reduction of DNT nitro group(s) to amino group(s), although the structure of the resulting fluorescent product was not independently confirmed. The Zn/HCl-treated product exhibited intense fluorescence emission at 427 nm following excitation at 305 nm, enabling sensitive quantification of DNT. Under the optimized experimental conditions, the method displayed excellent linearity over the concentration range of 30.0–250.0 ng mL^−1^ (*r* = 0.9997), with limits of detection and quantification of 7.14 and 21.65 ng mL^−1^, respectively. The analytical performance was comprehensively optimized by investigating the variables influencing the reduction reaction and fluorescence response, while the fluorescence quantum yield of the formed derivative was also determined. The method was successfully validated and applied to spiked chicken meat, liver, kidney, fat, and skin samples, yielding satisfactory recoveries across the investigated concentration levels. The selected spiking levels were evaluated in relation to the reported maximum residue limits (MRLs) for DNT in the corresponding poultry tissues. Furthermore, the environmental sustainability of the proposed assay was comprehensively assessed using the Multi-color Assessment (MA) Tool, the Analytical Green Star Area (AGSA), the Green Analytical Procedure Index (GAPI), and Analytical GREEnness (AGREE) metric, all demonstrating excellent green analytical performance. Combining operational simplicity, favorable analytical sensitivity, and satisfactory accuracy and precision, the proposed method offers an accessible alternative for determining DNT residues in poultry-derived food products. Although the results support its potential applicability to the investigated poultry matrices, further studies are warranted to strengthen its suitability for routine residue monitoring.

## Abstract 2

The first spectrofluorimetric method for dinitolmide residue analysis in poultry tissues, based on Zn/HCl treatment, offers high sensitivity, satisfactory recoveries, and excellent environmental sustainability.

## Graphical abstract

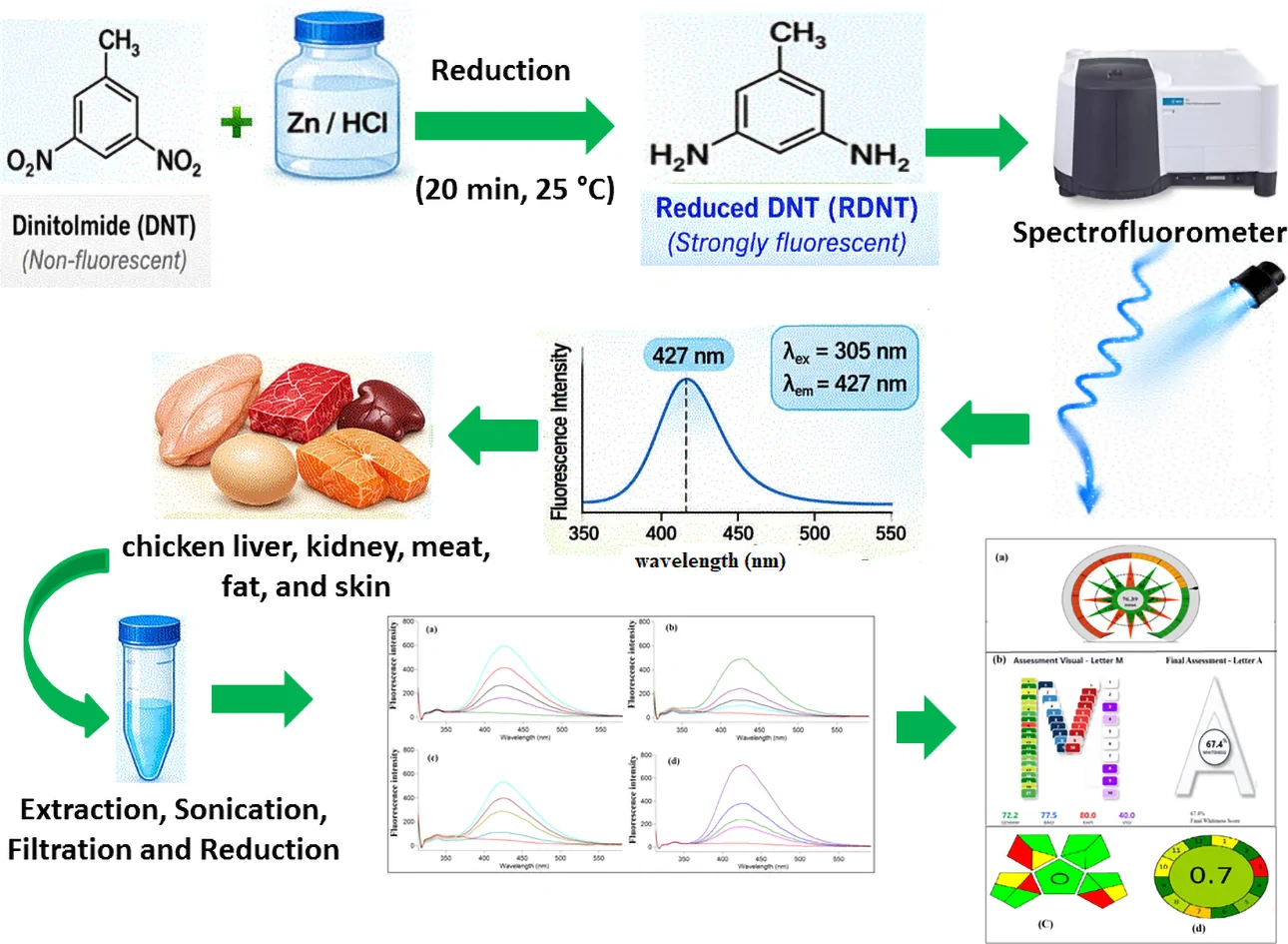

## 1. Introduction

Coccidiosis is a prevalent intestinal parasitic disease caused by *Eimeria* species and represents a major threat to the economic sustainability of the poultry industry. The parasite proliferates within the intestinal epithelium, inducing severe tissue damage that may result in diarrhea, intestinal hemorrhage, and impaired nutrient absorption. These pathological effects ultimately lead to reduced weight gain, poor feed conversion efficiency, and increased costs associated with disease control and prevention.^1^ Consequently, coccidiostatic agents such as dinitolmide (DNT) are widely used in poultry production to control coccidial infections, playing a crucial role in improving bird health, growth performance, and overall productivity of the poultry sector.^1^

Chemically, DNT is 2-methyl-3,5-dinitrobenzamide, as illustrated in Fig. 1.^2^ Owing to its high chemical stability, relatively low toxicity, and low propensity for the development of drug resistance, DNT is commonly incorporated into animal feed. Nevertheless, its prolonged and excessive use has resulted in the accumulation of toxic residues in poultry tissues, posing potential risks to human health through food consumption.^3^ Moreover, excessive administration leads to the excretion of the drug, either in its unchanged form or as metabolites, thereby contributing to environmental contamination and raising additional ecological concerns.^4^ Therefore, the development of rapid and highly sensitive analytical methods for the detection of dinitolmide is of paramount importance to ensure food safety and protect human health from potential exposure.

**Fig. 1:**
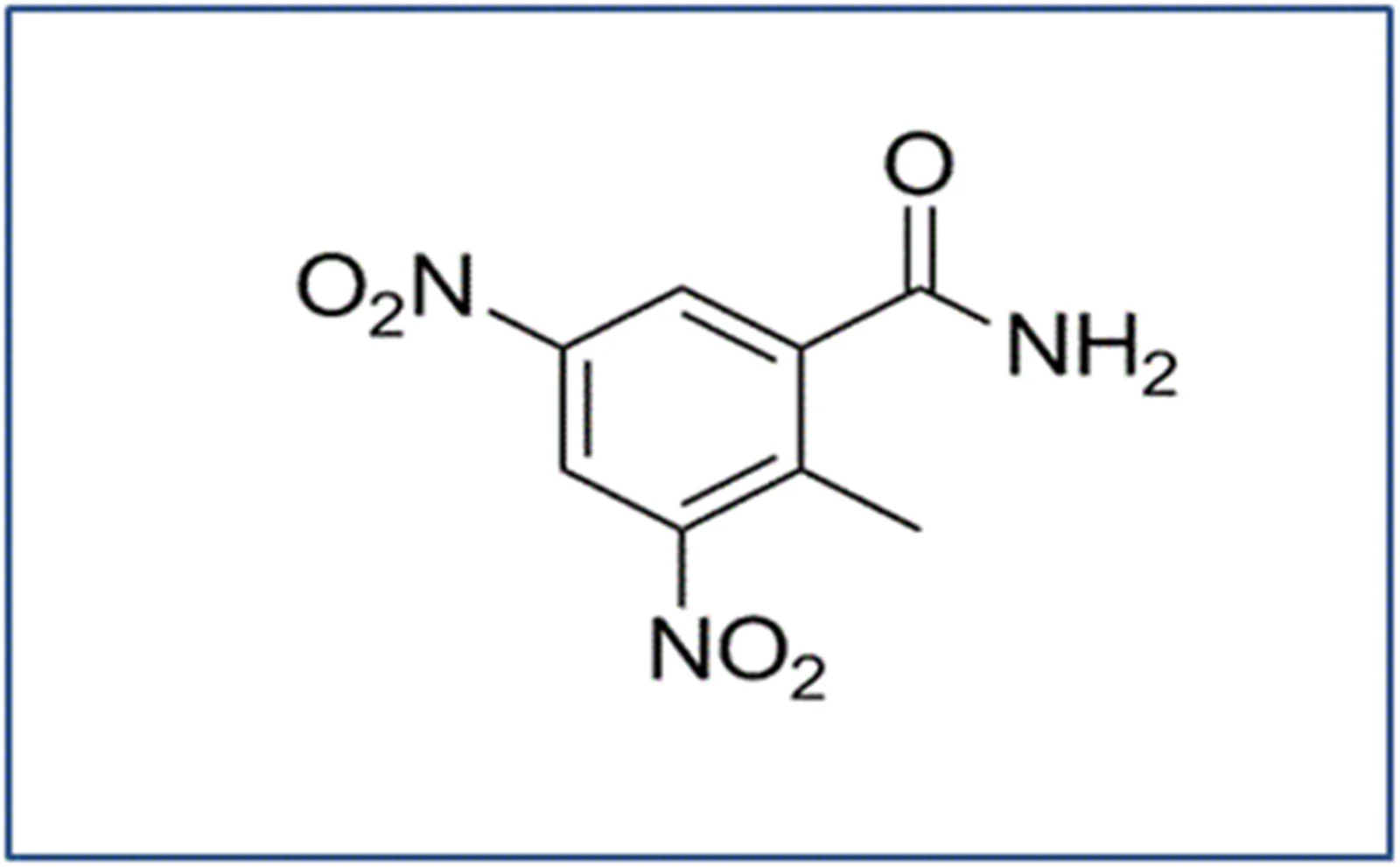
Structural formulae of DNT.

Accordingly, regulatory authorities have established maximum residue limits (MRLs) for DNT in animal-derived food products. The Chinese Ministry of Agriculture^5^ and the U.S. Food and Drug Administration^6^ have specified MRLs of 2000 µg kg^−1^ in fat and skin, 6000 µg kg^−1^ in liver and kidney, and 3000 µg kg^−1^ in chicken muscle. In addition, DNT residues are not detectable in eggs.

Commonly used analytical techniques for detecting DNT include electrochemical method,^7^ high-performance liquid chromatography (HPLC),^8^ high-performance liquid chromatography tandem mass spectrometry (LC-MS/MS),^9,10^ ultra performance liquid chromatography (UPLC),^11–14^ gas chromatography,^15,16^ surface-enhanced Raman spectroscopy (SERS),^17^ immune assay method,^18^ and Capillary electrophoresis (CE).^8^ Most of the reported analytical methods for DNT determination require extensive sample pretreatment, the use of sophisticated instrumentation, and hazardous reagents, rendering them neither cost-effective nor safe for routine application in quality control laboratories. Moreover, these methods are often time-consuming, limiting their suitability for high-throughput analysis. To the best of our knowledge, no spectrofluorometric method has yet been reported for the determination of DNT, either in its bulk form or as residues in animal-derived food products.

Spectrofluorimetric techniques possess several intrinsic advantages, such as outstanding sensitivity and selectivity, straightforward operation, rapid response, and broad accessibility in academic and research settings. These attributes have enabled their widespread use for the quantitative analysis of a wide range of pharmaceutical compounds in dosage forms and biological matrices.^19–28^ Owing to their capability for trace-level detection, spectrofluorimetric methods allow accurate quantification at nanogram concentrations, making them particularly well suited for pharmaceutical analysis in complex biological samples.^29–31^ Furthermore, spectrofluorimetry is regarded as a rapid, user-friendly, and environmentally sustainable analytical approach, as it generally involves minimal use of hazardous organic solvents and requires low energy consumption. Its non-destructive nature further enhances its applicability for the analysis of sensitive analytes within complex matrices.^32–38^

In the present study, a simple and sensitive spectrofluorometric method was developed and validated for the determination of trace levels (ng mL^−1^) of DNT residues in chicken meat, liver, kidney, fat, and skin samples. The method is based on the fluorescence response observed after treatment of DNT with Zn/HCl. This response is consistent with the proposed reduction of DNT nitro group(s) to the corresponding amino group(s), a reaction pathway that is chemically plausible given the known reducing behavior of Zn/HCl.^39^ However, fluorescence measurements alone cannot establish the molecular structure of the product, and no independent structural characterization was performed in the present study. For operational convenience, the fluorescent product obtained after Zn/HCl treatment is referred to as RDNT throughout this manuscript; this designation should not be interpreted as independent structural confirmation.

## 2. Experimental

### 2.1. Reagents and materials

Dinitolmide (DNT) was obtained from J&K Scientific Ltd (Beijing, China), while zinc powder was purchased from BDH Chemicals (UK) with particle size less than 50 µm. All other chemicals and reagents, including methanol, ethanol, acetonitrile, acetone, hydrochloric acid (37% w/v), phosphoric acid, sodium hydroxide, boric acid, glacial acetic acid, and sulfuric acid, were of analytical grade and supplied by Sigma-Aldrich (St. Louis, MO, USA). Carboxymethyl cellulose (CMC), β-cyclodextrin, Brij 30, Tween 80, and sodium dodecyl sulfate (SDS) were prepared as aqueous solutions at a concentration of 1% (w/v). Britton–Robinson buffer solutions (0.02 M) were prepared over a pH range of 2–12 using double-distilled water throughout the study. Chicken meat, liver, kidney, fat, and skin samples were purchased from the local market (Gomla Market, Mansoura City, Dakahlia Governorate, Egypt) and used for residue analysis.

### 2.2. Instrumentation

Fluorescence measurements were carried out using an Agilent Technologies Cary Eclipse^®^ spectrofluorimeter equipped with a xenon flash lamp (Santa Clara, CA, USA). The instrument was operated in high-voltage mode of 800 V with a smoothing factor of 20.0, and both excitation and emission slit widths were set at 5 nm. pH measurements were performed using a Jenway 3510 pH meter (Jenway, UK). UVvis absorption spectra were recorded on a double-beam spectrophotometer (PG Instruments, UK). Sample filtration was conducted using 0.45 µm membrane filters (Phenomenex, USA). An ultrasonic bath (Model SS 101H 230, USA) was employed for sonication, while a centrifuge (Model 2-16P, Germany) and a vortex mixer (Model IVM-300p, Gemmy Industrial Corp., Taiwan) were used during sample preparation.

### 2.3. Stock solution

A DNT methanolic stock solution (1.0 mg mL^−1^) was prepared and stored under refrigerated conditions at 4 °C until use. The stability of the stock solution was evaluated over 14 days of refrigerated storage at 4 °C. The emission intensity of the freshly prepared stock solution was recorded as the initial reference response (day 0), and the same stock solution was reanalyzed at predetermined time points under the same experimental conditions. Three independent measurements (*n* = 3) were performed at each time point. Stability was assessed by comparing the measured emission intensity at each time point with the initial response recorded on day 0. The measured responses remained within the predefined acceptable variation of ±2% throughout the study period, indicating that the DNT stock solution remained stable for at least 14 days under the specified storage conditions.

### 2.4. General procedures

#### 2.4.1. Treatment of DNT with Zn/HCl

An aliquot of 5.0 mL of the DNT stock solution was transferred into a 250 mL conical flask, followed by the addition of 5.0 mL of distilled water, 0.5 mL of hydrochloric acid, and 0.50 g of zinc powder with particle size less than 50 µm. The mixture was thoroughly mixed and allowed to react for 20 min at room temperature (25 °C) with occasional shaking. The reaction mixture was then filtered into a 50 mL volumetric flask. The conical flask and filter paper were rinsed three times with 5.0 mL portions of distilled water, and the rinses were combined with the filtrate. Finally, the solution was diluted to volume with distilled water to obtain a solution of the Zn/HCl-treated DNT reaction product at a DNT-equivalent concentration of 100.0 µg mL^−1^. For convenience, this solution is referred to as RDNT in the following sections; the product's chemical structure was not independently characterized.

#### 2.4.2. Construction of calibration curve

Aliquots of the RDNT standard solution corresponding to DNT-equivalent concentrations of 30.0–250.0 ng mL^−1^ were transferred into a series of 10 mL volumetric flasks and diluted to volume with ethanol. The relative fluorescence intensity (RFI) was measured at an emission wavelength of 427 nm using an excitation wavelength of 305 nm. Blank solutions were prepared and measured under identical conditions, and their RFIs were recorded. A calibration curve was constructed by plotting the RFI values against the corresponding DNT concentrations, and the regression equation was subsequently obtained.

#### 2.4.3. Application to chicken meat, liver, kidney, fat and skin samples

Chicken meat, liver, kidney, and combined fat and skin samples (approximately 5.0 g each) were accurately weighed and thoroughly homogenized. Appropriate volumes of a DNT standard solution (100.0 µg mL^−1^) were added to spike the samples at different concentration levels. The spiking levels were 1000.0, 1500.0, 2000.0, and 2500.0 µg kg^−1^ for chicken meat; 300.0, 500.0, 1000.0, and 2000.0 µg kg^−1^ for liver; 500.0, 1000.0, 1500.0, and 2000.0 µg kg^−1^ for kidney; and 300.0, 500.0, 1000.0, and 2000.0 µg kg^−1^ for the combined fat and skin samples. Each spiking experiment was performed in triplicate (*n* = 3). The samples were vortex-mixed for 2 min, followed by the addition of 10 mL of methanol. The spiked samples were sonicated for 15 min and then centrifuged at 10 000 rpm for 10 min. The resulting supernatants were filtered through 0.45 µm syringe filters and collected in 100 mL conical flasks. An aliquot of each prepared extract was mixed with 5.0 mL of distilled water, followed by the addition of 0.5 mL of hydrochloric acid and 0.50 g of zinc powder. The mixture was allowed to react for 20 min at room temperature, with occasional shaking. The reaction mixture was subsequently filtered into a 50 mL volumetric flask and diluted to volume with ethanol. The resulting solutions were analyzed according to the procedure described in Section 2.4.2. The DNT residue concentrations in the samples were quantified using the corresponding calibration regression equation.

#### 2.4.4. Evaluation of the quantum yield of the produced fluorophore

The quantum yield (*Φ*) of the produced fluorophore (RDNT) was evaluated using the comparative method according to eqn (1):^40^

1 *Φ*_RDNT_ = *Φ*_St_ × (*F*_RDNT_/*F*_St_) × (*η*_RDNT_/*η*_St_)^2^ × (*A*_St_/*A*_RDNT_)

where (*Φ*) is the fluorescence quantum yield, (*F*) is the integrated fluorescence intensity measured at an excitation wavelength of 305 nm, (*A*) is the absorbance at the excitation wavelength, and (*η*) is the refractive index of the solvent. 2-Aminopyridine dissolved in 0.1 M H_2_SO_4_ was used as the reference fluorophore, with a reported quantum yield (*Φ*) of 0.60 at 305 nm.^41^ RDNT was dissolved in ethanol. Accordingly, the refractive indices used for the RDNT and reference solutions were 1.36 (ethanol) and 1.33 (0.1 M H_2_SO_4_), respectively. To minimize reabsorption effects, the absorbance values of both the reference (*A*_St_) and RDNT (*A*_RDNT_) solutions were maintained below 0.1.

### 2.5. Statistical analysis

All experiments involving replicate measurements were performed in triplicate (*n* = 3), unless otherwise stated. Results were expressed as the mean and standard deviation (SD), where applicable. Precision was assessed using the percentage relative standard deviation (% RSD), calculated as (% RSD = (SD/mean) × 100). Method accuracy was evaluated in terms of percentage recovery, calculated as the ratio of the experimentally determined concentration to the corresponding spiked concentration, multiplied by 100. Calibration data were evaluated by linear regression, and the correlation coefficient was used to assess linearity. The limits of detection (LOD) and quantification (LOQ) were calculated using the standard deviation of the regression-line intercept (*S*_a_) and the slope of the calibration curve (*b*), according to the equations LOD = (3.3*S*_a_/*b*) and LOQ = (10*S*_a_/*b*), respectively. Descriptive statistical parameters were used to evaluate the precision and accuracy of the proposed method.

## 3. Results and discussion

Treatment of DNT with the Zn/HCl system produced a reaction mixture with a strong fluorescence response. The proposed reduction of DNT nitro group(s) to amino group(s) may account for the observed response, but this assignment was not confirmed by independent structural characterization. The Zn/HCl-treated product, referred to operationally as RDNT, exhibited an apparent fluorescence quantum yield of 0.342 in ethanol and a maximum emission at 427 nm upon excitation at 305 nm (Fig. 2). The fluorescence observations establish the analytical response of the reaction mixture but do not, by themselves, prove the structure or the formation of a specific stable resonant structure. The quantum yield of RDNT was evaluated using the procedure described in Section 2.4.4 and determined to be 0.342 (34.2%) under the experimental conditions employed.

**Fig. 2:**
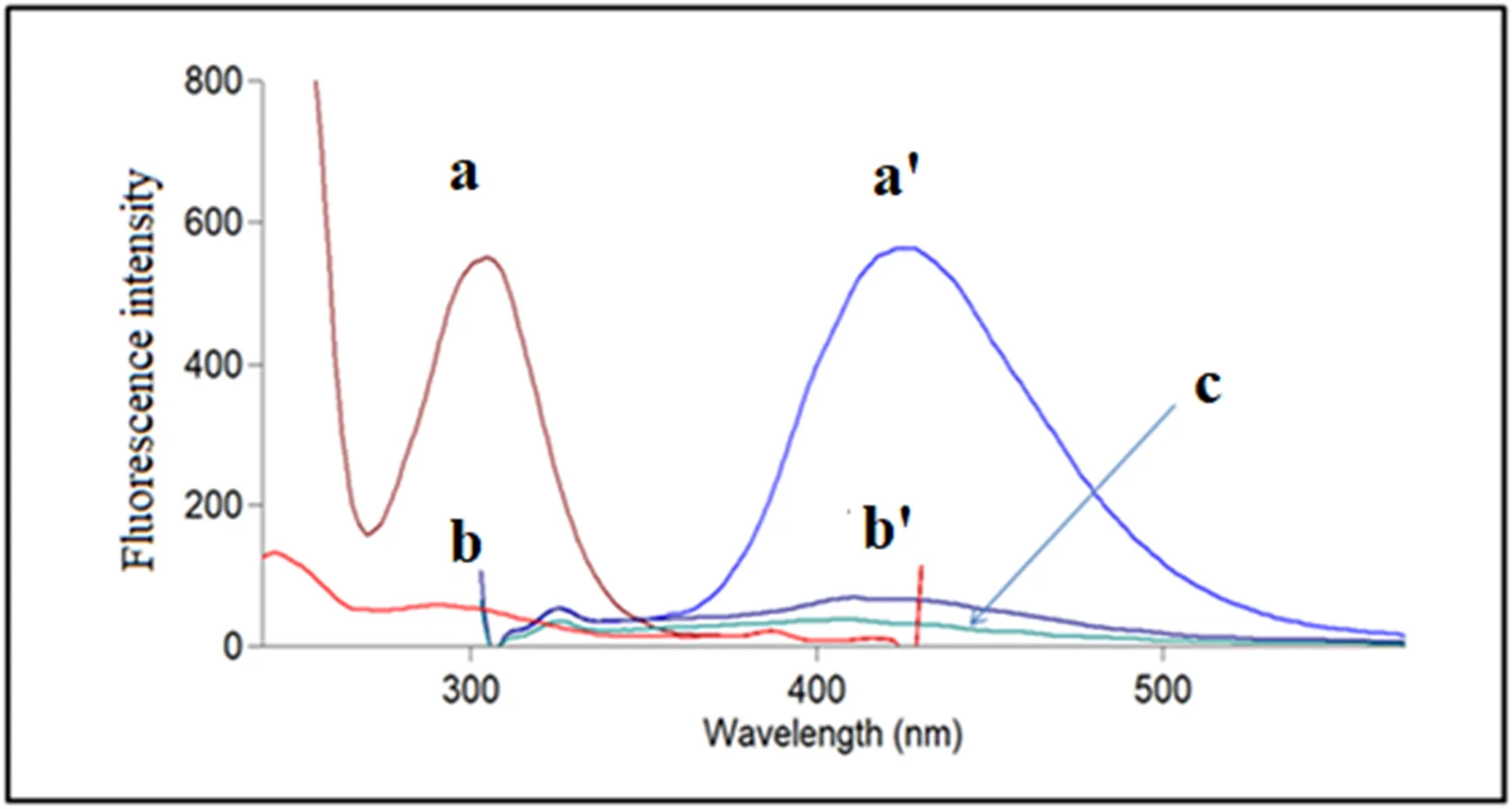
Emission (427 nm) and excitation spectra (305 nm) of the Zn/HCl-treated DNT solution (operationally denoted RDNT; 100.0 ng mL^−1^) (a and a′), ethanolic blank solution (b and b′) and emission spectrum of untreated DNT (100.0 ng mL^−1^) (c).

### 3.1. Optimization of experimental parameters

#### 3.1.1. Factors influencing DNT reduction

The influence of volume of HCl (0.25–2 mL) and Zn powder quantity (0.05–0.7 g) on the reaction product fluorescence intensity was studied to establish the optimal reduction conditions. Results showed that increasing the Zn amount to 0.5 g (Fig. 3a) and volume of HCl to 0.5 mL (Fig. 3b) enhanced the fluorescence, after which the values leveled off. Additionally, the fluorescent product development and stability was assessed by studying the influence of reduction time at room temperature (25 °C). The reduced form formed gradually, reached peak intensity after 20 minutes, and maintained stability for up to 2 hours.

**Fig. 3:**
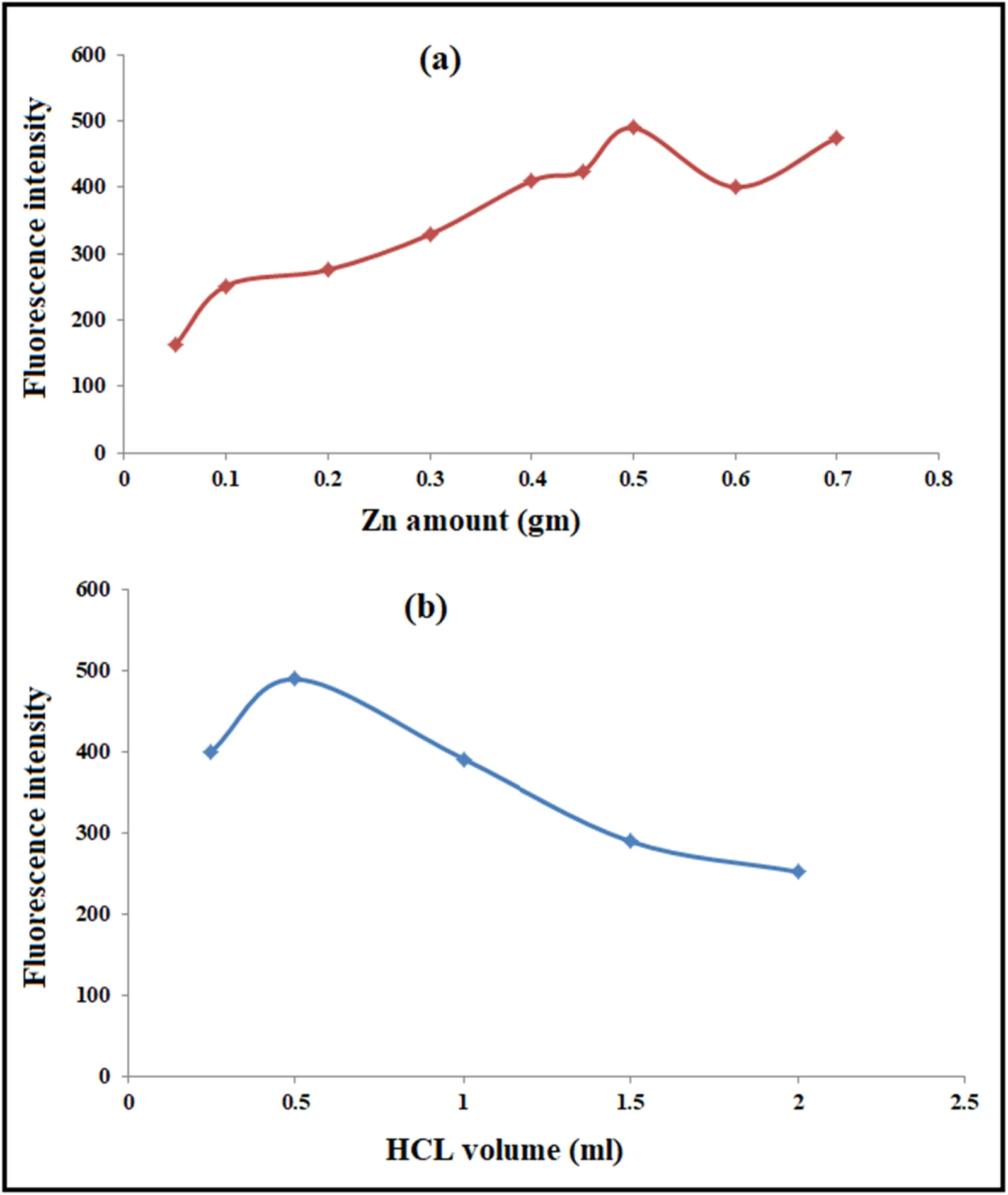
Effect of Zn powder quantity (0.05–0.7 g) (a) and HCl volume (0.25–2 mL) (b) on the fluorescence intensity of the reaction product.

#### 3.1.2. Effect of diluting solvent, pH, and organized media

The effect of different diluting solvents, namely methanol, acetonitrile, ethanol, acetone, and water, on the relative fluorescence intensity (RFI) of RDNT was investigated. Among the tested solvents, ethanol provided the highest and most reproducible RFI values. In contrast, water and acetonitrile caused a pronounced decrease in fluorescence intensity, while acetone resulted in significant fluorescence quenching (Fig. 4a). Accordingly, ethanol was selected as the optimal diluting solvent for subsequent experiments.

**Fig. 4:**
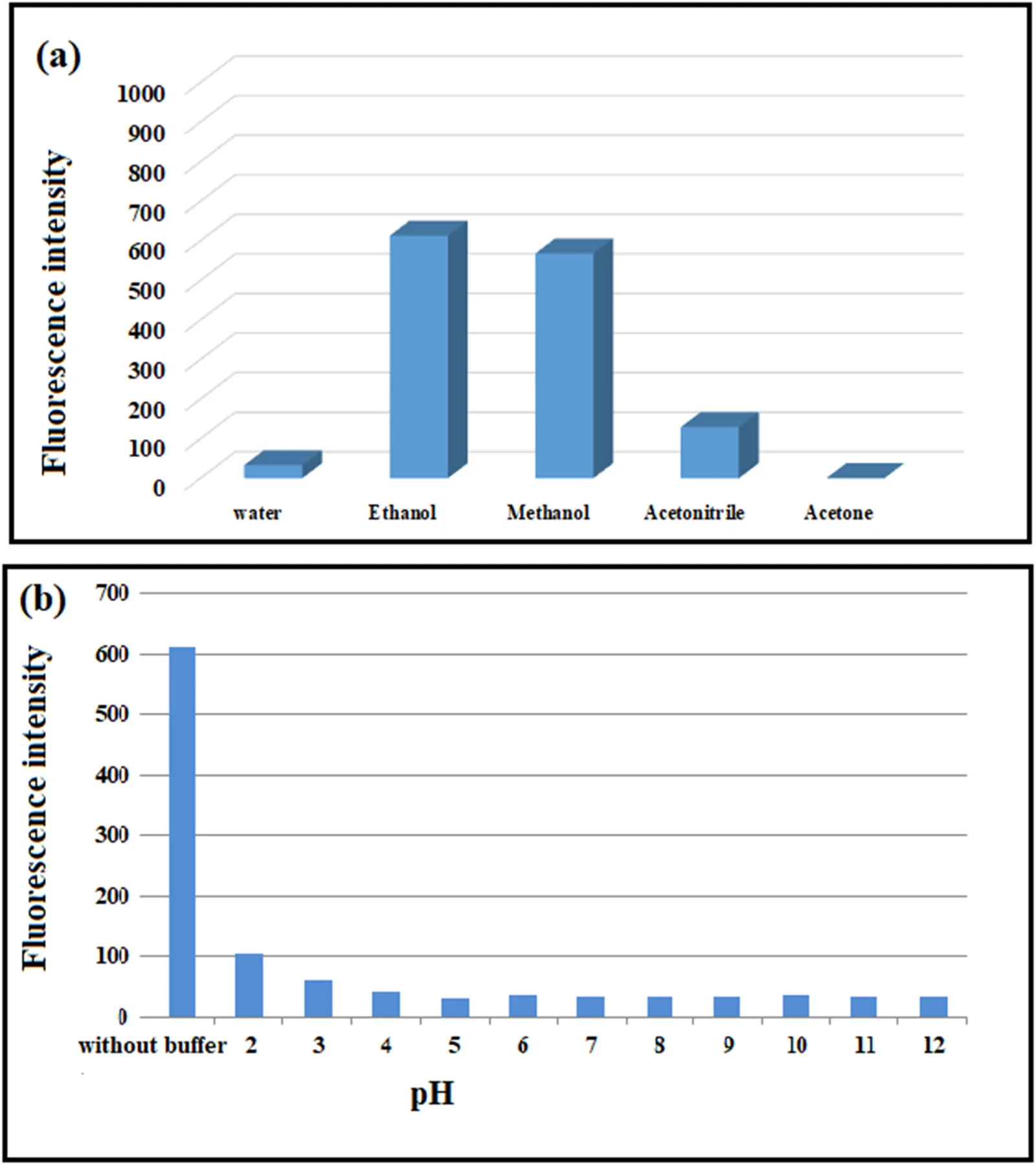
Effect of diluting solvents (a) and Britton–Robinson buffer (pH 2–12) (b) on the fluorescence intensity of the reaction product.

The fluorescence behavior of RDNT was further examined in Britton–Robinson buffer solutions over a pH range of 2–12 (Fig. 4b). Both acidic and alkaline media were found to markedly reduce the RFI of RDNT. Therefore, buffer solutions were not employed in the present study.

In addition, the influence of organized media, including surfactants such as β-cyclodextrin, sodium dodecyl sulfate, Tween-80, and Brij-30, as well as macromolecules like carboxymethylcellulose (CMC), was evaluated. All tested media led to a decrease in the RFI of RDNT (Fig. 5). Consequently, no organized media were used in the proposed method.

**Fig. 5:**
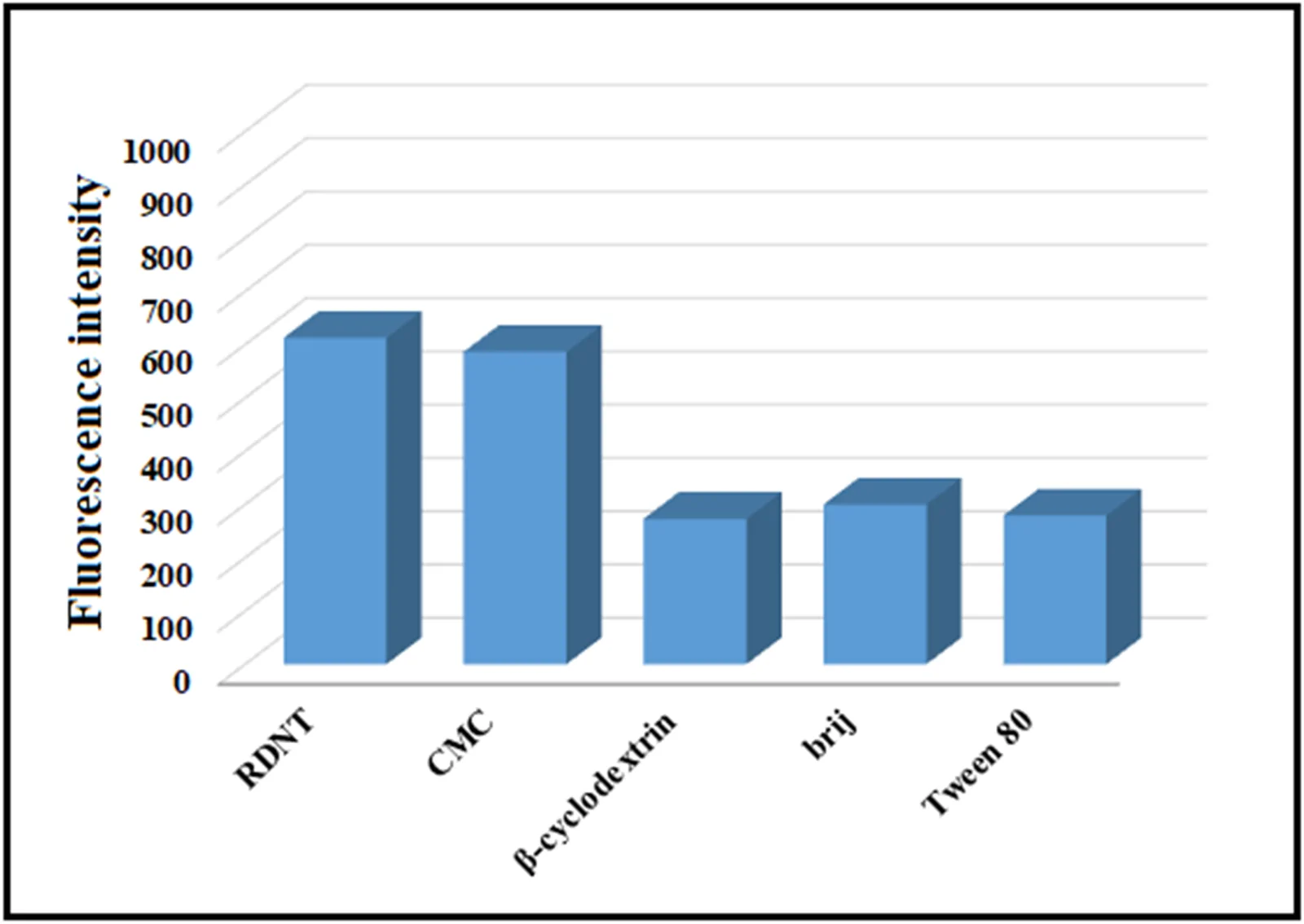
The influence of different surfactants on the fluorescence intensity of the reaction product.

### 3.2. Method validation

The validity of the newly suggested approach was verified in compliance with the ICHQ2 (R1) guidelines.^42^

#### 3.2.1. Range and linearity

The drug concentrations (ng mL^−1^) were plotted against the corresponding relative fluorescence intensity (RFI) values to construct the calibration curve, and the regression equation was subsequently calculated. Statistical evaluation of the data revealed low values of *S*_b_, *S*_*y*/*x*_, *S*_a_, and % RSD, along with a high correlation coefficient (*r*^2^), confirming excellent linearity of the calibration curve, as summarized in Table 1 and illustrated in Fig. 6. The method exhibited linearity over the concentration range of 30.0–250.0 ng mL^−1^ for DNT.

**Table 1:** Analytical performance data of the proposed method

Parameters	DNT
Wavelength (nm)	305/427
Linearity range (ng mL^−1^)	30.0–250.0
Limit of detection LOD (ng mL^−1^)	7.14
Limit of quantitation LOQ (ng mL^−1^)	21.65
Regression equation	RFI = 3.21*C* + 150.87
Correlation coefficient (*r*)	0.9997
S.D. of the residuals, *S*_*y*/*x*_	8.21
S.D. of the intercept, *S*_a_	6.94
S.D. of the slope, *S*_b_	0.043
Percentage relative standard deviation, % RSD	1.95
Percentage relative error, % error	0.87

**Fig. 6:**
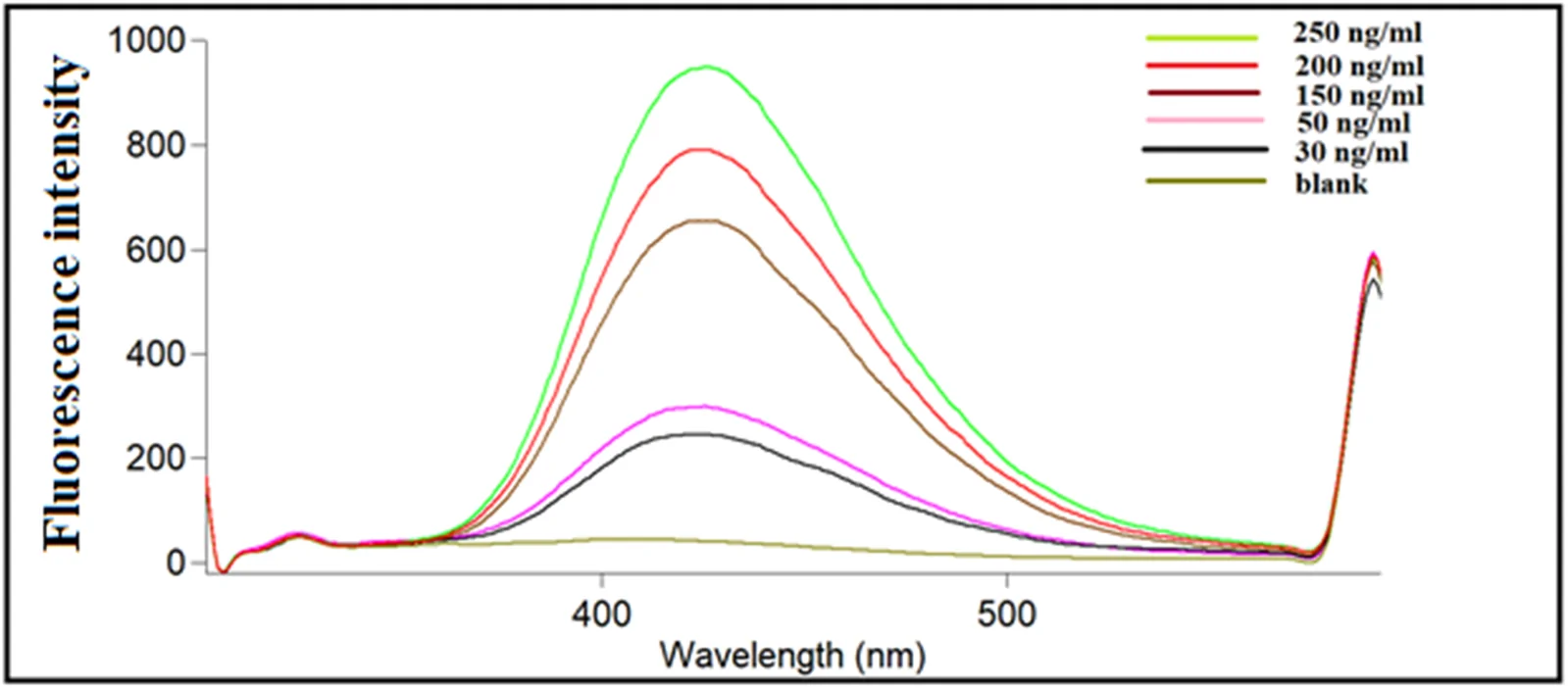
Fluorescence emission spectra of the Zn/HCl-treated DNT solution (operationally denoted RDNT) in ethanolic solution at DNT-equivalent concentrations of 30.0–250.0 ng mL^−1^.

#### 3.2.2. LOQ and LOD

The limits of detection (LOD) and quantification (LOQ) were determined and are summarized in Table 1. The following equations were used to calculate LOD and LOQ values:^43^

2 LOD = 3.3*S*_a_/*b*

3 LOQ = 10*S*_a_/*b*

where, *S*_a_ is the standard deviation of the intercept of a regression line and *b* is the slope of the linear calibration curve. The obtained results demonstrated the excellent sensitivity of the proposed method, confirming its suitability for the reliable quantification of the investigated drug in food samples.

#### 3.2.3. Precision and accuracy

The high recovery values obtained (97.27–102.58%) demonstrated the excellent accuracy of the proposed method for DNT determination, as summarized in Table 2. Moreover, analysis of the drug at three concentration levels, each performed in triplicate, yielded % RSD values below 2%, confirming the good intra- and inter-day precision of the method (Table 3).

**Table 2:** Application of the proposed method for the determination of DNT in standard solutions

Parameter	Proposed method		
	Conc. taken (ng mL^−1^)	Conc. found (ng mL^−1^)	% Recovery^a^
DNT	30.0	29.6218	98.74
	50.0	48.6368	97.27
	150.0	153.8737	102.58
	200.0	199.3600	99.68
	250.0	248.4999	99.40
Mean			99.53
±SD			1.94
% RSD			1.95
% Error			0.868

a Mean of three determinations (*n* = 3).

**Table 3:** Intra-day and inter-day precision data for the determination of DNT by the proposed method

Analyte	Conc. taken (ng mL^−1^)	Intra-day			Inter-day		
		Conc. found^a^ (ng mL^−1^) ± S.D.	% RSD	% Error	Conc. found^a^ (ng mL^−1^) ± S.D.	% RSD	% Error
DNT	30.0	29.95 ± 0.90	0.91	0.53	29.99 ± 1.85	1.85	1.07
	150.0	149.94 ± 0.47	0.47	0.27	149.96 ± 0.31	0.31	0.18
	200.0	199.75 ± 0.24	0.25	0.14	199.81 ± 0.04	0.04	0.02

a Each result is the average of three separate determinations (*n* = 3).

#### 3.2.4. Robustness

To evaluate the robustness of the proposed method, small deliberate variations were introduced into the experimental conditions, and the consistency of the recorded relative fluorescence intensity (RFI) values was examined. The investigated variations involved key parameters affecting the reduction of DNT, namely the amount of zinc powder (0.50 ± 0.05 g) and the volume of hydrochloric acid (0.50 ± 0.10 mL). These minor, intentional changes did not produce any significant effect on the RFI of RDNT, confirming the robustness of the method (Table 4).

**Table 4:** Robustness evaluation of the proposed method

Variation	DNT	
	% Recovery	% RSD
**1 – Zn powder (0.50 ± 0.05 g)**		
0.45 g	100.98	1.20
0.50 g	100.91	0.56
0.55 g	101.28	0.48

**2 – Volume of HCL (0.50 ± 0.1 mL)**		
0.40 mL	101.08	0.94
0.50 mL	100.91	0.56
0.60 mL	100.83	1.19

**3 – Reduction time (20 ± 1 min)**		
19 min	99.91	1.50
20 min	100.91	0.56
21 min	100.35	0.86

#### 3.2.5. Selectivity

The selectivity of the developed method was evaluated by investigating potential interference from co-administered drugs, including amprolium and amoxicillin. The respective tolerance limits were 10.0 and 5.0 µg mL^−1^, indicating that these compounds did not significantly interfere with DNT determination under the investigated conditions. Selectivity was further assessed using different poultry tissue matrices, including chicken meat, liver, kidney, fat, and skin. For each matrix, a corresponding unfortified sample was subjected to the same sample preparation and Zn/HCl reduction procedures without DNT fortification. The fluorescence response of each matrix blank was subtracted from that of the corresponding spiked sample to correct for background fluorescence, and the corrected responses were used for quantitative analysis.

The applicability of the proposed method was further evaluated using spiked poultry tissue samples at different concentration levels. The mean recoveries were 100.26% for chicken meat, 99.93% for liver, 100.00% for kidney, and 100.01% for fat and skin, with corresponding % RSD values of 3.560%, 1.768%, 0.837%, and 4.891%, respectively (Table 5). These results demonstrate satisfactory recovery and precision, supporting the applicability of the method to the investigated matrices under the experimental conditions employed. Nevertheless, blank correction and satisfactory recovery results do not necessarily exclude all potential matrix effects, particularly those that may affect analytical sensitivity.

**Table 5:** Estimation of DNT in food samples by the proposed method

	Final conc. (ng mL^−1^)	Conc. found (ng mL^−1^)	Spiked conc. (µg kg^−1^)	Volume taken (µL)	% Recovery^a^
**Chicken meat**					
	100.0	103.7048	1000.0	50.0	103.70
	150.0	148.6975	1500.0	75.0	99.13
	200.0	191.5018	2000.0	100.0	95.75
	250.0	256.1057	2500.0	125.0	102.44
Mean ± SD					100.26 ± 3.57
% RSD					3.560
Regression equation	RFI = 2.8698*C* − 136.8320				

**Liver**					
	30.0	29.4281	300.0	15.0	98.09
	50.0	51.1388	500.0	25.0	102.28
	100.0	99.2667	1000.0	50.0	99.27
	200.0	200.1709	2000.0	100.0	100.09
Mean ± SD					99.93 ± 1.77
% RSD					1.768
Regression equation	RFI = 2.3225*C* + 27.4532				

**Kidney**					
	50.0	50.1437	500.0	25.0	100.29
	100.0	99.0422	1000.0	50.0	99.04
	150.0	151.4811	1500.0	75.0	100.99
	200.0	199.3295	2000.0	100.0	99.66
Mean ± SD					100.00 ± 0.84
% RSD					0.837
Regression equation	RFI = 2.8189*C* − 28.4900				

**Fat and skin**					
	30.0	28.7371	300.0	15.0	95.79
	50.0	53.3703	500.0	25.0	106.74
	100.0	97.0938	1000.0	50.0	97.09
	200.0	200.8027	2000.0	100.0	100.40
Mean ± SD					100.01 ± 4.89
% RSD					4.891
Regression equation	RFI = 3.2233*C* + 68.1716				

a Mean of three determinations (*n* = 3).

The effects of common pharmaceutical excipients, including lactose, maltose, mannitol, dextrin, and citric acid, were also investigated as a complementary assessment of potential formulation-related interferences. These compounds exhibited negligible effects on the fluorescence intensity of RDNT (Fig. 7a). In addition, the tested metal ions (Na^+^, K^+^, Ca^2+^, Mg^2+^, and Ba^2+^) did not significantly interfere with DNT determination under the investigated conditions (Fig. 7b).

**Fig. 7:**
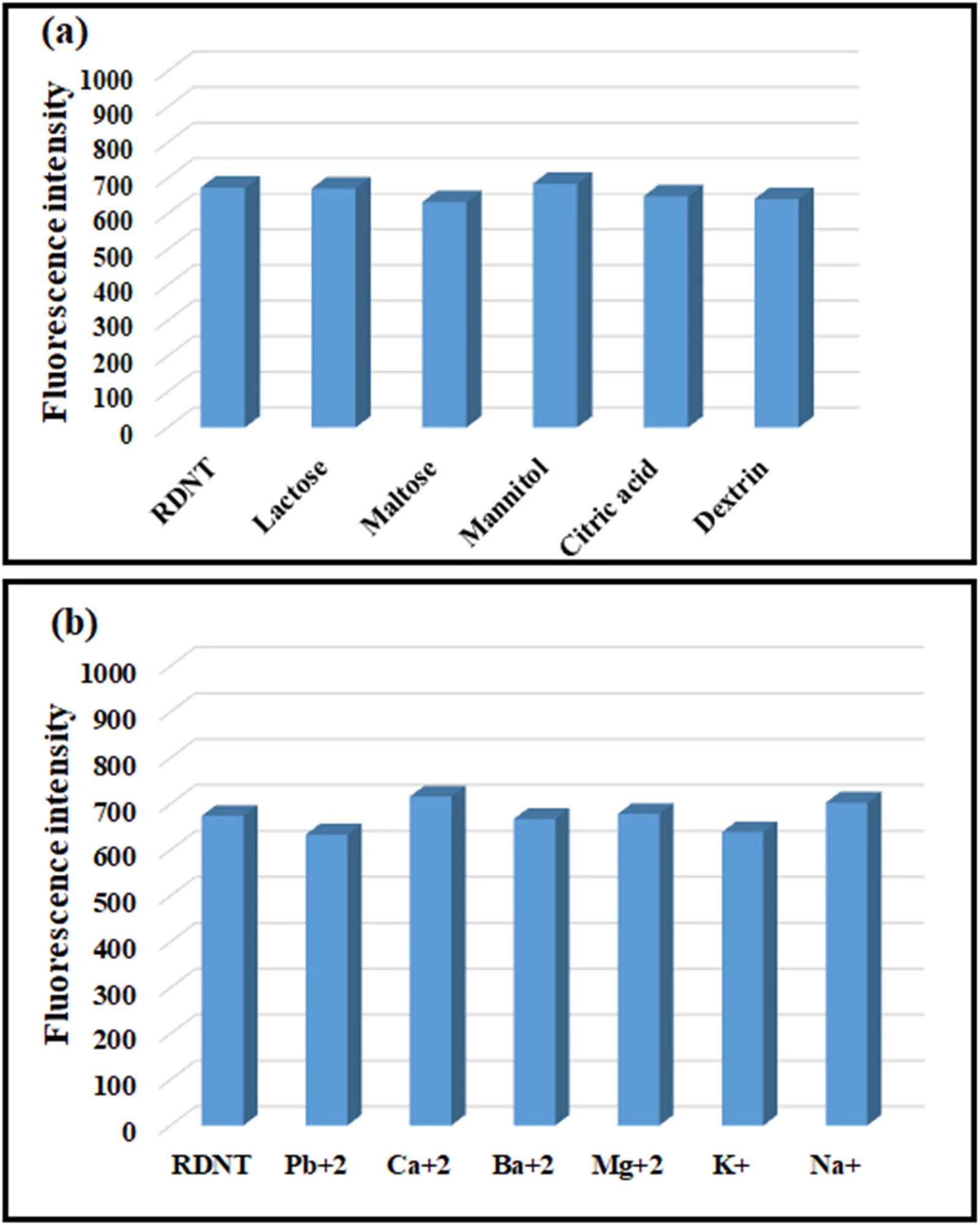
Selectivity of the developed method for DNT determination using the fluorescence response of the Zn/HCl-treated DNT solution (operationally denoted RDNT) in the presence of possible interfering excipients (a) and different metal ions (b).

Although amprolium did not significantly affect the fluorescence response attributed to DNT in the interference experiment at its tolerance limit of 10.0 µg mL^−1^, its fluorescence response following Zn/HCl treatment was not independently characterized. Therefore, its possible reduction or formation of fluorescent products under these conditions cannot be definitively excluded. Similarly, the individual contributions of endogenous matrix components, particularly proteins and lipids, were not separately investigated. These limitations should be considered when interpreting the selectivity results.

### 3.3. Applications

#### 3.3.1. Determination of DNT in food samples

The applicability of the proposed method was evaluated using spiked chicken meat, liver, kidney, fat, and skin samples. The nominal spiking concentrations were selected with reference to the MRLs for DNT reported in the cited regulatory sources: 3000 µg kg^−1^ for chicken muscle, 6000 µg kg^−1^ for liver and kidney, and 2000 µg kg^−1^ for fat and skin.^5,6^ The investigated spiking levels ranged from 1000.0 to 2500.0 µg kg^−1^ for chicken meat, 300.0 to 2000.0 µg kg^−1^ for liver, 500.0 to 2000.0 µg kg^−1^ for kidney, and 300.0 to 2000.0 µg kg^−1^ for fat and skin. Thus, the tested levels were below the cited MRLs for chicken meat, liver, and kidney, whereas the highest tested level for fat and skin was equal to the cited MRL. These comparisons refer to the nominal concentrations used in the spiking experiments and do not establish the residue status of naturally contaminated samples. The results obtained for the spiked samples are presented in Table 5 and Fig. 8. The proposed method exhibited satisfactory performance, with recovery values ranging from 95.75 to 106.74%, supporting its analytical applicability to the investigated poultry matrices. Although the recovery results demonstrate satisfactory performance in spiked poultry matrices, the present experiments do not establish the method's ability to determine regulatory compliance in naturally contaminated samples. Furthermore, because the proposed fluorescence response may include contributions from DNT-related compounds, the results should be interpreted in accordance with the method's selectivity limitations and the applicable regulatory residue definition. Confirmation of individual residues and regulatory compliance may require a suitably validated confirmatory method, such as LC-MS/MS.

**Fig. 8:**
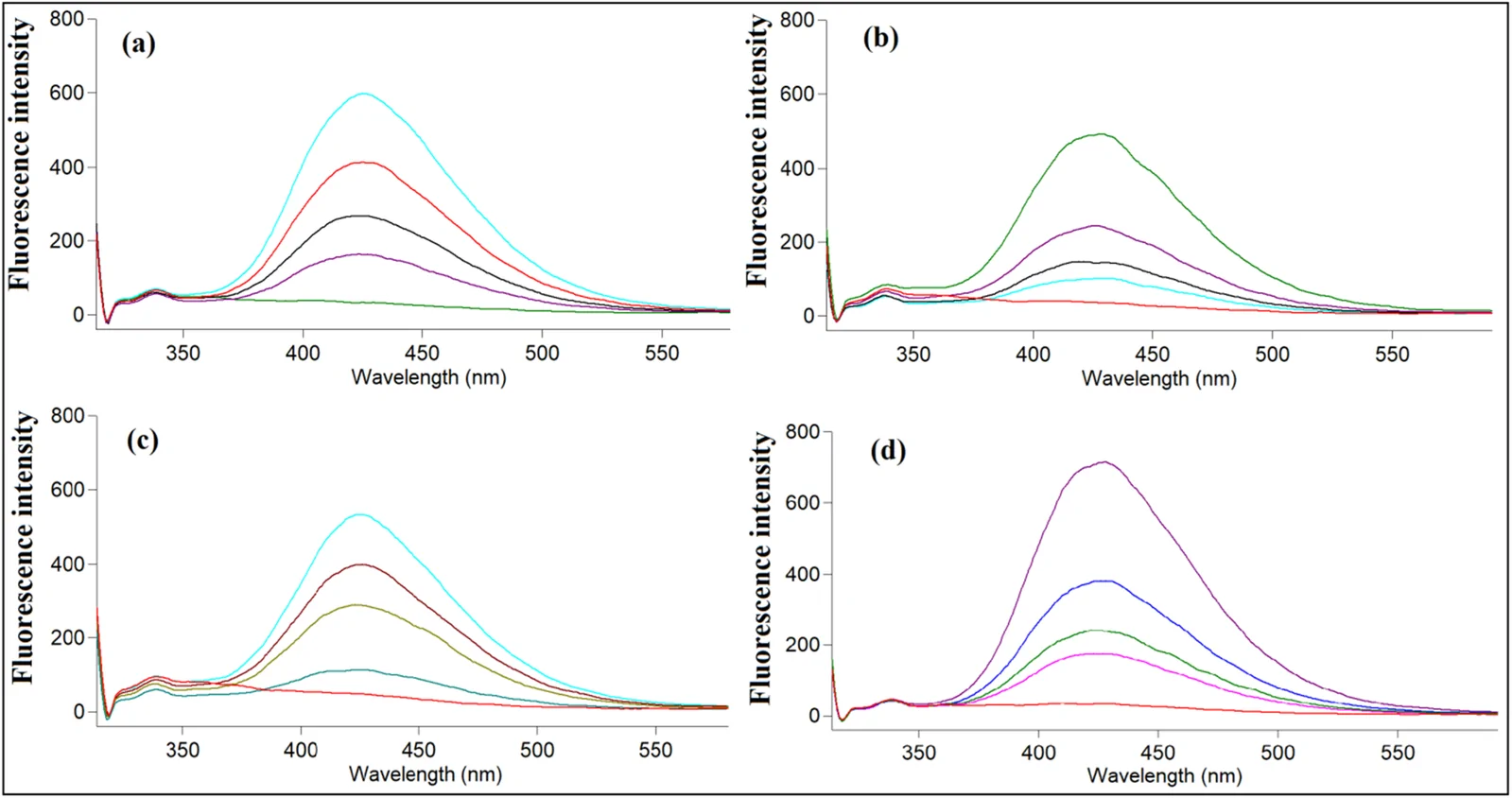
Fluorescence emission spectra obtained during DNT determination in food samples at different DNT-equivalent concentrations: (a) meat (from top to bottom: 100, 150.0, 200.0, 250.0 ng mL^−1^), (b) liver (from top to bottom: 30, 50.0, 100.0, 200.0 ng mL^−1^), (c) kidney (from top to bottom: 50, 100.0, 150.0, 200.0 ng mL^−1^), and (d) combined fat and skin (from top to bottom: 30, 50.0, 100.0, 200.0 ng mL^−1^).

#### 3.3.2. Toxicological relevance and metabolite detection

From a food safety and human health perspective, exposure assessment involves not only the parent drug (DNT) but also its primary metabolic derivatives. In poultry, DNT undergoes *in vivo* biotransformation predominantly *via* partial reduction of a nitro group to yield 3-amino-5-nitro-*o*-toluamide (3-ANOT).^15,16^ Both DNT and its reduced metabolite 3-ANOT contribute to toxic residue burdens in poultry products. Because the proposed Zn/HCl reduction system targets the nitro group functionality present in both parent DNT and its nitro-containing metabolites, the resulting fluorescence response reflects the total chemical residue bearing nitro moieties. This non-selective chemical conversion enables the proposed spectrofluorimetric method to function as an efficient and rapid screening platform for total DNT-related residue load. However, because the method measures cumulative fluorescence without chromatographic separation, it cannot differentiate parent DNT from 3-ANOT. Consequently, samples testing positive during routine spectrofluorimetric screening should undergo subsequent hyphenated chromatographic analysis (*e.g.*, LC-MS/MS or GC-MS/MS) when individual metabolite speciation and regulatory quantification against specific single-compound MRLs are required.^4,15,16^

### 3.4. Evaluation of method's greenness and sustainability by different metric tools

Due to the increasing concern about environmental sustainability, various tools and metrics have been developed to assess the environmental impact of different analytical techniques.^44–46^ Despite the fact that each reported tool has its unique computation and criteria, these metrics and tools may encourage chemists to adopt environmentally friendly practices.^47^ Therefore, scientists may be able to determine which is more appropriate for their goals and the level of accuracy they require from these tools.^48^ Analytical Green Star Area,^49^ MA Tool,^50^ the Green Analytical Procedure Index (GAPI),^51^ and Analytical GREEnness (AGREE)^52^ are metrics we used to evaluate the sustainability of the proposed approach in this context.

To assess entire analytical process, the AGSA was recently developed.^49^ By offering both quantitative and visual insights on the environmental performance of analytical techniques, the AGSA metric offers a robust framework for evaluating their greenness. The 12 principles of green analytical chemistry (GAC) can be assessed utilizing this new metric, which also offers a visual depiction of the overall environmental impact. It assures a thorough assessment of analytical techniques by emphasizing efficiency and safety as well as reducing waste, energy use, and toxicity. In Fig. 9a, the analysis results of AGSA for the suggested method is displayed. Four reported evaluation models; GEMAM, BAGI, RAPI, and VIGI, are combined into a single, all-inclusive system for assessing analytical techniques by the MA Tool, an innovative and interactive digital platform. By measuring performance, originality, practicality, and greenness and combining them into a single Whiteness Score. Fig. 9b shows the MA Tool analysis results, confirming the method's excellent greenness and sustainability profiles.

**Fig. 9:**
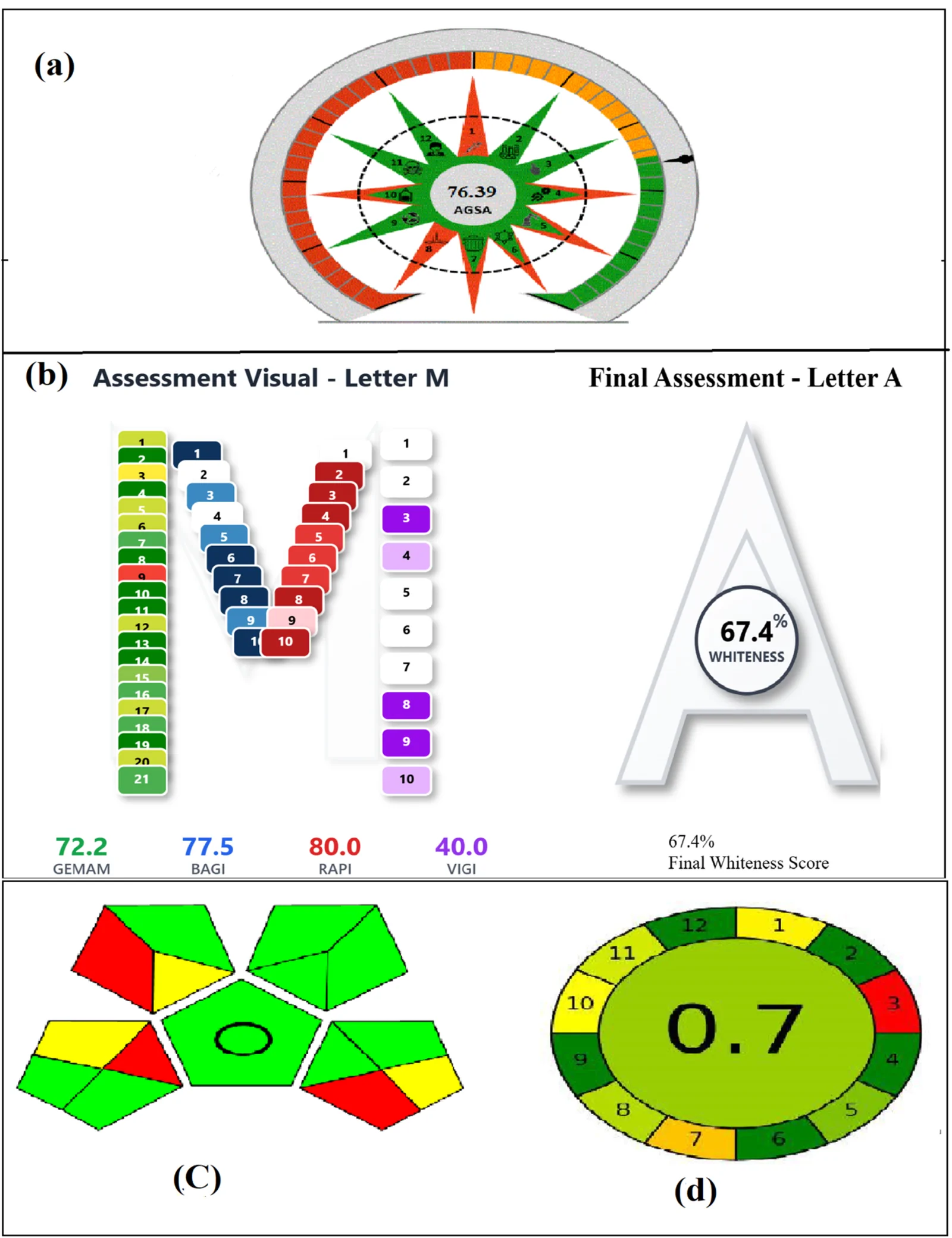
Assessment of the greenness and sustainability profile of the proposed method by (a) AGSA, (b) MA Tools, GAPI (c), and AGREE (d).

The GAPI was recently created to assess the whole analytical process. The five pentagrams on it stand for technique type, sample collection and preparation, chemicals and solvents, and tools. Green denotes a step with no impact on the environment, yellow denotes a step with a moderate environmental impact, and red denotes a potential environmental risk. The green potential of the suggested approach was demonstrated using GAPI, from the sample collecting stage to the completion. The suggested procedure's GAPI pictogram is shown in Fig. 9c. The sole negative is that the protein precipitation step of the food samples pretreatment procedure using a little amount of methanol. Additionally, the procedure's environmental friendliness was also evaluated using metric tool called AGREE, that emphasizes sample preparation. It is built upon 12 impact categories, which were divided into sub scores on a zero to one scale and utilized to determine the evaluation's ultimate outcome. The selection and use of solvents, materials, and reagents, as well as the throughput, energy usage, waste production, and sample size were some of the evaluation criteria. The evaluation also made use of the capacity to distinguish between the application of criteria by assigning them weights. The outcomes of the AGREE analysis are shown in Fig. 9d. On a scale of zero to one, the suggested method's AGREE analysis had a general score of 0.7, demonstrating low environmental effect and the acceptable greenness of the method.

### 3.5. Comparison with previously reported methods

The analytical performance and practical applicability of the proposed spectrofluorimetric method were compared with those of previously reported methods for DNT determination (Table 6). Regarding analytical sensitivity, the proposed method exhibited an LOD of 0.00714 µg mL^−1^, which was lower than the reported values for the electrochemical method (0.1611 µg mL^−1^),^7^ HPLC-DAD method (0.243 µg mL^−1^),^8^ surface-enhanced Raman spectroscopy (SERS) method (0.03 µg mL^−1^),^17^ capillary electrophoresis-DAD method (1.010 µg mL^−1^),^8^ and immunoassay method (0.00901 µg mL^−1^).^18^ These comparisons indicate favorable detection limits relative to several reported techniques, although differences in analytical matrices and experimental procedures should be considered when interpreting the results.

**Table 6:** Comparison of the proposed spectrofluorimetric method with previously reported methods for the determination of DNT

Technique	Matrix	Sample preparation/complexity	Linearity range	LOD	Cost	Speed of analysis	Reference
Electrochemical method	Pure and food samples	Protein precipitation and requires functionalized electrode preparation	0.543–3.255 µg mL^−1^	0.1611 µg mL^−1^	Low cost; specialized electrode materials required	Fast	7
HPLC-DAD	Pure and pharmaceutical dosage forms	No sample preparation	1–15 µg mL^−1^	0.243 µg mL^−1^	Moderate instrumental and solvent costs	4.3 min	8
LC-MS/MS	Food samples	High; complex multi-step cleanup/solid-phase extraction required	0.075–6 µg kg^−1^	0.067 µg kg^−1^	High equipment, operational, and solvent costs	3 hours	10
UPLC-MS	Food samples	Liquid–liquid extraction	0.001–0.03 µg mL^−1^	—	High equipment cost	Long analysis run time	12
GC-MS/MS	Eggs	High; accelerated solvent extraction (ASE) and solid-phase extraction	1.0–100 µg kg^−1^	0.3 µg kg^−1^	High equipment cost	Long analysis run time	15
Surface-enhanced Raman spectroscopy	Chicken meat	Protein precipitation	0.1–10 µg mL^−1^	0.03 µg mL^−1^	Moderate instrument cost	Fast	17
Immune assay method	Chicken meat	Protein precipitation	0.00316–0.02565 µg mL^−1^	0.00901 µg mL^−1^	High	15 min for strip readout	18
Capillary electrophoresis – DAD	Pure and pharmaceutical dosage forms	No sample preparation	5–50 µg mL^−1^	1.010 µg mL^−1^	High	2.97 min	8
Spectrofluorimetry	Pure, chicken meat, liver, kidney, fat, skin	Protein precipitation	0.03–0.25 µg mL^−1^	0.00714 µg mL^−1^	Very low cost; accessible spectrofluorimeter & high greenness	20 min	This method

From an instrumentation perspective, the proposed method relies on conventional spectrofluorimetric equipment and therefore avoids the need for sophisticated mass spectrometric instrumentation required by LC-MS/MS,^10^ UPLC-MS,^12^ and GC-MS/MS.^15^ It also avoids the specialized electrode fabrication and modification required by the reported electrochemical method.^7^ For poultry tissue samples, the proposed procedure involves methanolic extraction followed by Zn/HCl reduction, providing an alternative approach to methods that employ more elaborate extraction and cleanup procedures. These features may facilitate implementation in laboratories with limited access to advanced analytical instrumentation.

As summarized in Table 6, the proposed method was also applied to multiple poultry tissue matrices, including chicken meat, liver, kidney, fat, and skin. Its practical advantages include accessible instrumentation, a relatively straightforward sample preparation procedure, and favorable detection limits compared with several reported methods. However, the overall analysis time and operational cost depend on the complete analytical workflow, including sample preparation, measurement, and instrument requirements. Accordingly, the proposed method represents a potentially accessible alternative for DNT determination in the investigated poultry matrices, while the choice of analytical technique should take into account the intended application and the specific performance requirements.

### 3.6. Limitations of the study

Although the proposed spectrofluorimetric method offers a sensitive, simple, and relatively straightforward approach for screening DNT residues, several limitations should be acknowledged. The observed fluorescence response following Zn/HCl treatment is consistent with the proposed reduction of DNT nitro group(s) to amino group(s), based on the known reducing behavior of this reagent system.^39^ However, no independent structural elucidation of the reaction product using mass spectrometry, NMR, IR spectroscopy, or elemental analysis was performed. Accordingly, the product is described operationally as RDNT, and its precise chemical identity and the proposed reaction pathway remain to be confirmed by appropriate structural analyses. In addition, the method was evaluated using in vitro-spiked tissue matrices. Although satisfactory recoveries were obtained across the investigated chicken tissues, *in vitro* spiking may not fully replicate *in vivo* drug deposition, cellular entrapment, or tissue–protein binding following therapeutic administration. Furthermore, spectrofluorimetry lacks the inherent separation capability of chromatographic techniques; therefore, samples requiring definitive identification or quantification of individual residues or metabolites should be examined using a suitable confirmatory method, such as LC-MS/MS. Application to other matrices with high intrinsic background fluorescence may also require additional cleanup steps (*e.g.*, solid-phase extraction).

## 4. Conclusion

A sensitive, simple, and rapid spectrofluorimetric method was developed and validated for the determination of DNT in poultry tissue matrices. The method is based on the fluorescence response generated following treatment of DNT with Zn/HCl. The observed response is consistent with the proposed reduction of nitro group(s) to amino group(s); however, because the reaction product was not independently characterized, the proposed mechanism and the precise chemical identity of the product remain provisional. The Zn/HCl-treated reaction product, operationally designated as RDNT, exhibited an apparent fluorescence quantum yield of 0.342 under the investigated conditions. The method demonstrated satisfactory analytical performance in spiked chicken meat, liver, kidney, fat, and skin samples, with acceptable recovery and precision. Furthermore, amprolium and amoxicillin did not significantly interfere with DNT determination within their respective tested tolerance limits. Nevertheless, the independent fluorescence response of amprolium following Zn/HCl treatment and the individual contributions of endogenous matrix components, particularly proteins and lipids, were not fully characterized and warrant further investigation. The environmental sustainability of the proposed method was evaluated using complementary greenness assessment tools, including AGSA, AGREE, GAPI, and the MA Tools. Overall, the method shows promise for the analysis of DNT in the investigated poultry matrices under the studied conditions. Further characterization of the reaction product and additional selectivity studies, together with validation at relevant regulatory residue limits and confirmatory analysis where required, would strengthen its suitability for routine residue monitoring.

## Conflicts of interest

The authors declare that they have no known competing financial interests or personal relationships that could have appeared to influence the work reported in this paper.

## Data Availability

The datasets generated and/or analyzed during the current study are available from the corresponding author on reasonable request.
